# Protective Effect of *Tulbaghia violacea* Harv. on Aortic Pathology, Tissue Antioxidant Enzymes and Liver Damage in Diet-Induced Atherosclerotic Rats

**DOI:** 10.3390/ijms131012747

**Published:** 2012-10-08

**Authors:** Olubukola S. Olorunnisola, Graeme Bradley, Anthony J. Afolayan

**Affiliations:** 1 Department of Biochemistry and Microbiology, University of Fort Hare, Private Bag X1314, Alice 5700, South Africa; E-Mails: sinbadkd@gmail.com (O.S.O.); GBradley@ufh.ac.za (G.B.); 2 Phytomedicine Research Group, Department of Botany, University of Fort Hare, Alice 5700, South Africa

**Keywords:** *T. violacea* rhizomes, anti-atherosclerosis, lipid derangements, endothelium dysfunction, antioxidant enzymes

## Abstract

The protective effect *Tulbaghia violacea* rhizomes (TVR) against derangements in serum lipid profile, tissue antioxidant enzyme depletion, endothelium dysfunction and histopathological changes in the aorta and liver of rats fed with an atherosclerogenic (Ath) diet (4% cholesterol, 1% cholic acid and 0.5% thiouracil) was investigated in this study. Co-treatment with the TVR extracts (250 and 500 mg/kg body weight for two weeks significantly (*p* < 0.05) protected against elevated serum triglyceride (TG), total cholesterol (TC), LDL-cholesterol, VLDL-cholesterol and decreased HDL-cholesterol in a dosedependent manner when compared with the atherogenic control. The extracts also reduced (*p <* 0.05) elevated thiobabutric reacting substance (TBARS) and reversed endothelial dysfunction parameters (fibrinogen and total NO levels) and tissue antioxidant enzyme activities to near normal. The protective ability of the extract was confirmed by the significant (*p <* 0.05) reduction in the activities of serum markers of liver (LDH, AST, ALT, ALP, bilirubin) and kidney damage (creatinine and bilirubin) in extract-treated groups compared with the atherogenic control group. Also, histopathology evaluations of aorta sections revealed that the extracts protected against the development of fatty streak plaques (aorta) and fatty changes in hepatocytes. The observed activities of the extracts compared favorably with standard drug atorvastatin. Our study thus showed that the methanolic extract of TVR could protect against the early onset of atherosclerosis.

## 1. Introduction

Atherosclerosis is a multifactorial disease of the large and medium-sized muscular arteries and the leading cause of morbidity and mortality in industrialized countries [1]. It is characterized by endothelial dysfunction, vascular inflammation, and build-up of lipids, cholesterol [2], calcium and cellular debris within the intima of the vessel wall [3]. This leads to build-up in plaque formation, vascular remodeling, acute and chronic luminal obstruction, abnormalities of blood flow and diminished oxygen supply to target organs [4]. Depending on the location of the blocked arteries, atherosclerosis may lead to complications generally referred to as cardiovascular diseases (coronary artery disease, carotid artery disease, peripheral artery disease, aneurysms, heart attack and stroke). Sandra and Lewis [5] reported that successful treatment minimizes lifetime chances of cardiovascular events, morbidity, and mortality. It was suggested that risk factors for atherosclerosis should be monitored, beginning in childhood, even in asymptomatic patients. Modifiable factors (e.g., blood pressure, smoking, serum lipids) and non-modifiable factors should be assessed (e.g., age and family history) [5]. Improved lifestyle such as dietary choices, increased exercise, and smoking cessation combined with lipid-lowering pharmacotherapy and antihypertensive medication may also be employed in the management and prevention of atherosclerosis [5]. However, the treatment and management of this disease is still a challenge to the medical system [6]. Though a large number of hypolipidemic drugs are currently available in the market, they lack desired properties such as efficacy and safety of long term use, cost and simplicity of administration [7]. These deficiencies have led to an increase in the demand for an inexpensive, affordable medication without any adverse side effects. Recently, attention has been focused on a number of medicinal plants used in the treatment of cardiovascular disease because of their reported lipid lowering, anti-anginal, antioxidant and cardioprotective effect. One of the commonly used plants in the Eastern Cape of South Africa for the treatment of heart disease is the rhizomes of *Tulbaghia violacea* Harv. [8].

*Tulbaghia violacea* is a fast-growing, bulbous plant that reaches a height of 0.5 m. It was reported to possess many biological activities such as *in vitro* antithrombotic activity [9] *in vitro* antioxidant activity [10], Antibacterial activities against *Staphylococcus aureus* and *Bacillus subtilis* [11], *in vitro* anticancer [12] and anthelmintic activity [13]. It is believed that *T. violacea* may possess biological activities similar to garlic (*Allium sativum*) since both belong to the same Alliaceae family [14]. However, most of the biological activities demonstrated by garlic remain to be investigated in *T. violacea*.

In the present study, we have for the first time investigated the effect of an extract of *T. violacea* rhizomes on markers of endothelial dysfunction, lipid profile and tissue antioxidant status in diet-induced atherosclerogenic rats.

## 2. Results and Discussion

Protection against arterial endothelial injuries such as the development of fatty streak plaques and in-vessel-wall cholesterol accumulation may prevent atherosclerosis. Hyperlipidemia and oxidative stress have been reported to play a critical role in endothelial dysfunction and most chronic diseases such as atherosclerosis [15]. Food intake, body weight, and water consumption were similar for all the groups during the two weeks study (Table 1). High cholesterol content (cholesterol 1%) in atherogenic diets has been reported to lead to increased body weight in experimental rats [16]. The results of the present study are consistent with the above observation (Table 1). Co-administration of the extracts (250 mg/kg and 500 mg/kg) and the standard drug (atorvastatin: 30 mg/kg) significantly reduced (*p <* 0.05) the increase in body weight induced by an atherogenic diet. The results suggest that the extract may be employed in weight control.

Our results (Table 2) in the present study revealed that rats on an atherogenic diet showed a significant (*p <* 0.05) increase in serum TC, TG, LDL-c, VLDL-c and a significant decrease in HDL-c compared with normal rats. Our observation was consistent with the reports of Azonov *et al.* [17] and Sunder *et al.* [18]. Elevated serum cholesterol, TG, and VLDL-c, LDL-c and decreased HDL-c have been implicated in the etiology of cardiovascular diseases (CHD) [19]. High serum lipids (TG, and VLDL-c, LDL-c) contributed to the development of cardiovascular diseases in various ways. According to Gokkusu and Mostafazadeh, [20] hypercholesterolemia increases aortic thiobabutric reacting substance (TBARS) or malondialdehyde and oxygen radicals, resulting in endothelial cell injury, modulation of cell adhesion molecules and eventually the development of atherosclerosis [21].

Co-treatment of methanolic extracts of *Tulbaghia violacea* rhizomes (TVR) along with an atherogenic diet significantly (*p <* 0.05) reduced serum total cholesterol (TC), triglyceride (TG), low density lipoprotein (LDL) and very low density lipoprotein (VLDL) in a dose dependent manner. The anti-hyperlipidemia activity of the extract was highest at 500 mg/kg bwt. The LDLs are highly atherogenic lipoproteins [22]. They are primary carriers of plasma cholesterol. Oxidation of LDL in the walls of arteries may lead to impaired endothelium-mediated relaxation in isolated arterial segments thereby causing atherosclerosis and increasing the risk of high blood pressure, which may eventually lead to stroke [23]. The reduction in LDL-c levels in the extract-treated group when compared with the atherogenic negative control may lead to enhanced protection against LDL oxidation, offering an alternative mechanism for the observed antiatherogenic property of the plant. High density lipoprotein (HDL) is reported to possess anti-atherogenic properties [24], HDL is involved in the transport of cholesterol from peripheral tissues to the liver and thereby reduces the amount stored in the tissue and the possibility of developing atherosclerotic plaques [23]. Reduction in blood cholesterol has been reported to reduce vascular resistance by improving endothelial function [24]. It also inhibits the oxidation of LDL by virtue of its antioxidant and anti-inflammatory properties [25]. In this study, HDL was significantly (*p* < 0.05) increased in a dose-dependent manner in the extract-treated groups when compared with the atherogenic control (Table 1). Similar observations have been reported for two species of garlic (*Allium sativum* and *Allium turberosum*) [26]. This indicates that TVR may help to increase the transport of peripheral tissue cholesterol to the liver and thereby decrease blood cholesterol when concomitantly fed with an atherogenic diet. The atherogenic index ratio (TC –HDL-C/HDL-C) is a powerful indicator of the risk of cardiovascular disease [27–29]. The higher the value (≥ 4.5), the higher the risk of developing cardiovascular disease and *vice versa* [24]. In this study, we observed that the TVR significantly (*p <* 0.001) reduced atherogenic indices in a dose dependant manner (Table 2). A lower atherogenic index ratio (≤ 3) indicates protection against coronary heart disease [30]. The antilipidemia activity of TVR extract may be attributed to the inhibitory effect of its organosulfur constituents [10].

The anti-lipidemia activity of the extract compared favorably with the standard drug (atorvastatin) especially at the highest concentration of 500 mg/kg). The triacylglycerol-lowering effect of the TVR extract is an added advantage. Elevated triglyceride has been identified as an independent risk factor for cardiovascular disease [24]. Hypertriglyceridaemia is frequently caused by elevated VLDL levels. The anti-triglyceridemia effect may be due to the extract/atrovastatin ability to inhibit fatty acid synthesis and some metabolic enzymes such as fatty acid synthetase and glucose-6-phosphate dehydrogenase [31].

**Figure 1:**
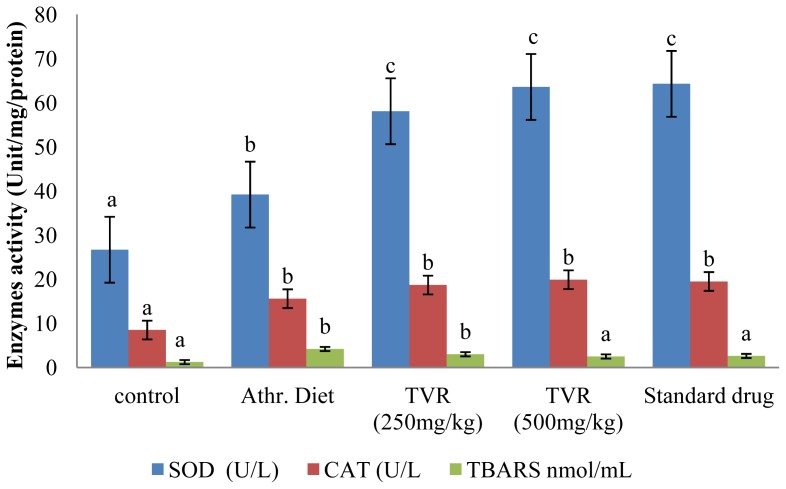
Effect of various doses of TVR on activity of tissue antioxidant enzymes in arota of diet-induced atherogenic rats. Mean values with the same letter subscripts within the different group are not significantly different (*p* < 0.05).

**Figure 2:**
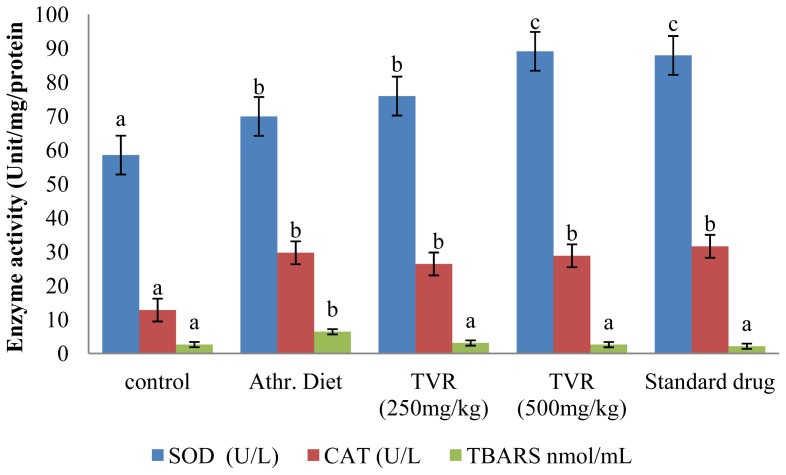
Effect of various doses of TVR on activity of tissue antioxidant enzymes in liver of diet-induced atherogenic rats. Mean values with the same letter subscripts within the different group are not significantly different (*p* < 0.05).

The ability of the TVR to protect against aortic, liver antioxidant enzyme depletion and endothelial dysfunction was also investigated. The results (Figures 1 and 2) revealed a significant (*p <* 0.05) increase in the activity of superoxide dismutase (SOD), catalase (CAT) and TBARS levels in both aorta and liver of the diet-induced atherosclerogenic control group compared with the animals on a standard diet. The increase in TBARS formation is consistent with observations reported by Yanling *et al.* [32]. However, the elevation of both hepatic and aortic antioxidant enzyme activities (SOD and CAT) in this study contradicts their reports (Figures 1 and 2). The increase in antioxidant enzyme activities may be a result of an early response or resistance to oxidative insults and lipid peroxidation occasioned by the atherogenic diet.

Co-administration of the extract (250 and 500 mg/kg bwt) and atorvastatin (30 mg/kg bwt) along with the atherogenic diet significantly (*p* < 0.05) increased the enzyme activities (SOD and CAT) in both hepatic and aortic tissues but caused a significant reduction (*p <* 0.05) in the level of TBARS in the TVR group compared with the atherogenic control group (Figures 1 and 2).

The increase in antioxidant enzyme activities in the extract-treated groups may be due to the ability of the extract to activate enzyme synthesis. Our results also demonstrate that the methanolic extract of TVR may protect the liver and aorta against atherogenic induced oxidative stress. The protective potential of the extract was further supported by a significant (*p* < 0.05) reduction in the levels of platelet, fibrinogen and an increase (*p* < 0.05) in the level of nitric oxide (NO) in extract treated groups compared with the atherogenic control (Table 3). The activity of the extract was comparable to the ameliorating effect of the standard drug (atorvastatin) on endothelia dysfunction.

Increased serum platelet, fibrinogen and decreased endothelial nitric oxide have been reported in endothelial dysfunction and the development of atherosclerosis [33]. Platelets play a major role in vascular wall homeostasis and etiology of atherosclerosis [34]. They may adhere to exposed sub-endothelium after endothelial injury and release vasoactive substances that induce smooth muscle cell migration and proliferation [35], development of fatty streaks by serving as a lipid source [36] or promote foam cell formation [37]. Platelets may release many substances that can induce further platelet accumulation and activation, vasoconstriction, thrombosis, and mitogenesis, including ADP, serotonin, platelet-derived growth factor, fibroblast growth factor, ADP, serotonin, platelet factor 4, β-thromboglobulin [38,39] and superoxide anions [40], all of which play a significant role in the development and progression of atherosclerosis. The reduced platelet concentration in the extract-treated groups compared with the atherogenic control showed that the extract can protect against atherogenic diet-induced endothelial damage. The anti-platelet properties of the extract may be due to its polyphenol content [40]. Pignatelli *et al.* [41] reported that polyphenol inhibits platelet NADPH oxidase in a protein kinase C (PKC) dependent manner in addition to its free radical scavenging properties.

Nitric oxide is a potent vasodilator, an inhibitor of platelet aggregation, smooth muscle cell proliferation and adhesion of monocytes to endothelial cells [42].

**Figure 3:**
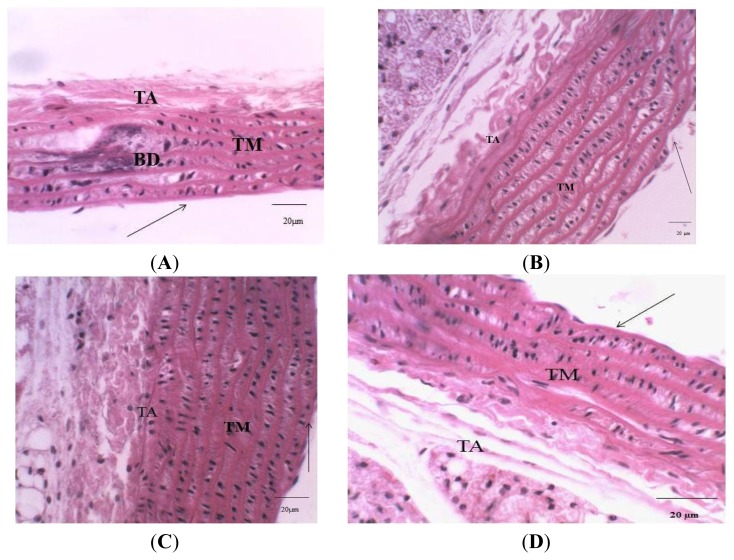
Photomicrograph of rat aorta. (**A**) The aorta of atherogenic rat; (**B**) Control aorta of rat; (**C**) The aorta of (atherogenic diet +500 mg/kg of RTV) rat; (**D**) The aorta of (atherogenic diet + 30 mg/kg of atorvastatin) rat. Showing straight tunica intimae (thin- dark arrow); Tunica media (TM) with elastic fibers, basophilic changes (BD) and tunica adventitia (TA). Scale bar: 20 μm, H & E stain, 400× magnification).

Endothelial damage occasioned by atherogenic diet-induced oxidative insults might result in the destabilization of endothelial enzymes generating NO (endothelial nitric oxide synthase (eNOS)) leading to a reduced NO production and endothelial dysfunction [43] The observed insignificant difference in the nitric oxide and the fibrinogen concentration in the extract-treated groups compared with the atherogenic control may be connected to the antioxidant, antilipidemic and antiplatelet properties of the TVR which protect the endothelium against atherogenic diet-induced damage. In addition, the extract may share a similar mechanism of action with the drug atorvastatin, which has been reported to prevent hypoxia-induced down regulation of eNOS in endothelial cells by stabilizing eNOS MRNA leading to an increased NO production [44]. Histopathological evidence (Figure 3C) revealed that the extracts protect against swelling of the fibers in the wall or foam cell formation in the aorta by inhibiting mononuclear cell adhesion, their emigration into the intima and an accumulation of lipids in the vessel wall.

The protective potential of extracts of TVR against atherogenic diet-induced liver damage was also assessed in the present study. Table 4 reveals that all serum markers of liver or kidney damage were elevated in rats fed with an atherogenic diet alone compared with animals on a standard rat diet. The observed increase in the serum marker enzymes of hepatic tissue of rats fed with an atherogenic diet is likely due to cellular damage caused by an atherogenic diet-induced steatohepatitis with cellular ballooning via cholesterol-induced oxidative stress [45] which leads to lipid peroxidation of biomolecules and leakage of cellular components. Our results are in agreement with the report of Sunder *et al.* [18] in which they reported that an atherogenic diet caused a significant increase in the serum markers of liver (LDH, AST, ALT, ALP, bilirubin) and kidney damage (creatinine).

An atherogenic diet has been reported to induce glomerulosclerosis/nephropathy and mild tubular and hepatic damage experimental rats [18]. Co-treatment with TVR along with an atherogenic diet significantly protected against elevated serum markers of liver and kidney damage in a dose dependant manner. Our results revealed that the kidney and hepatoprotective properties of the extract compared favorably with the standard drug used as positive control in this study. The protection against kidney hepatic damage by the extract may be due to its phytochemical components such flavonoids and saponins [10] which have been reported to possess hepatoprotective effects [46,47].

## 3. Materials and Methods

### 3.1. Plant Collection and Extract Preparation

Plant collection and extract preparation followed the earlier description by Mohammad and Woodward [48] and was modified according to Olorunnisola *et al.* [10].

### 3.2. Animals

Healthy, female Wister albino rats (137–165 g) were randomly assigned to control and treated groups (six animals per group/cage). They were maintained in standard environmental conditions (22 ± 2 °C, 12 h:12 h dark/light cycle, humidity: 45%–50%). The animals were allowed to acclimatize to the environment for seven days and supplied with a standard pellet diet (Hindustan Lever Ltd., Bangalore India) and water *ad libitum*. Before induction of atherosclerosis, the weight of the individual animals and plasma cholesterol levels were determined. All animals were obtained from the animal house of the laboratory of the School of Biological Sciences, University of Fort Hare Alice, and South Africa. All procedures used in the present study followed the “Principles of Laboratory Animal Care” from NIH Publication No.85-23 and were approved by the Animal Ethics Committee of our university.

### 3.3. Experiment Design

The experimental animals were divided into five groups and an atherogenic diet was prepared according to the method described by Sunder *et al.* [18].

Group 1: Experimental animals fed with a standard diet and orally administered 1 mL distilled water served as control.
Group II: Negative control rats were fed with an atherogenic diet comprised of normal rat chow plus 4% cholesterol, 1% cholic acid and 0.5% thiouracil.
Group III and IV: These experimental animals were fed with an atherogenic diet comprised of the normal rat chow plus 4% cholesterol, 1% cholic acid and 0.5% thiouracil, but also supplemented orally with extract of *T. violacea* (0.25 g/kg and 0.50 g/kg body weight, respectively) once daily for two weeks.
Group V: These rats were fed with an atherogenic diet [normal rat chow plus 4% cholesterol, 1% cholic acid and 0.5% thiouracil) supplemented with standard atorvastatin orally (30 mg/kg body weight) suspended in distilled water once daily for two weeks.

### 3.4. Body Weight, Food Intake and Water Consumption

The body weight of the animals was measured at the initiation of the treatment, every week thereafter, and on the day of sacrifice. The food intake was measured in g/kg bwt/day at the start of the treatment and at weekly intervals thereafter. The amount of food was measured before being supplied to the cage and any food that remained the following day was also weighed.

### 3.5. Sample Collection

At the end of the two weeks, the overnight fasted rats were sacrificed under ether anesthesia. Blood samples were collected using vacuum tubes with/without EDTA from each rat for determination of hematological parameters and biochemical analysis, respectively. The blood sample collected in a plain tube was centrifuged at 3000 rpm for 10 min to obtain serum for biochemical analysis. Immediately after collecting blood, the liver and aorta from each rat were removed, and placed into clean and dry tubes.

### 3.6. Liver and Aorta Homogenate Preparation

Liver and aorta homogenates were prepared as described by Noori *et al.* [49]. Briefly, livers were perfused with saline and homogenized in chilled KCl (1.17%) using a homogenizer. The homogenates were then centrifuged at 800*g* for five minutes at 4 °C to get post mitochondrial supernatant. Whole aorta tissue was homogenized in KH_2_PO_4_ buffer (100 mM) containing EDTA (1 mM) (pH 7.4) (1:10 *w*/*v*) and centrifuged at 12,000× *g*, by 30 min, at 4 °C). The supernatant was used for biochemical estimations.

### 3.7. Hematological Analysis

The blood chemistry assay was carried out using Beckman Coulter^4C(R)^ (USA). Hematological parameters assayed for included fibrinogen, white blood cell (WBC), red blood cell (RBC) and differential leukocyte counts, pack cell per volume (PCV) and platelet counts (PT).

### 3.8. Biochemical Assay

Biochemical parameters, serum alkaline phosphatase (ALP), alanine aminotransferase (ALT), aspartate aminotransferase (AST), total bilirubin (TB), serum triglyceride (TG), total serum cholesterol (TC), high density lipoprotein cholesterol (HDL-c), low density lipoprotein (LDL-c) and very low density lipoprotein (VLDL-c) were measured using a Piccolo automated chemistry analyzer (Abaxis, Inc. Union City. CA, USA). NO production was determined by measuring the accumulation of nitrite in the aorta supernatant. Nitrite in aorta supernatants was measured, as described by Green *et al.* [50], by adding 100 μL of Griess reagent (1% sulfonamide and 0.1% *N*-w1-naphthylx-ethylenediamine in 5% phosphoric acid) to 100 μL supernatant from samples and the mixture was incubated for 10 min at room temperature. The OD at 550 nm was measured using a Spectra max micro-plate reader (Molecular Devices, Bio Tek, and USA). The nitrite concentration in micromole was calculated from a sodium nitrite standard curve. Tissue antioxidant enzyme activities were also monitored in the liver and aorta supernatant.

### 3.9. Determination of Catalyse Activity (CAT)

Catalyse activity was measured as described by Pari and Latha [51]. Briefly, the tissue was homogenized in 0.01 M phosphate buffer (pH 7.0) and centrifuged at 5000 rpm. The reaction mixture consisted of 0.4 mL of hydrogen peroxide (0.2 M), 1 mL of 0.01 M phosphate buffer (pH 7.0) and 0.1 mL of liver homogenate (10% *w*/*v*). The reaction of the mixture was stopped by adding 2 mL of dichromate-acetic acid reagent (5% K_2_Cr_2_O_7_) prepared in glacial acetic acid). The changes in the absorbance were measured at 620 nm and recorded. The percentage of inhibition was calculated using the Equation:

(1) % catalase inhibition=[(normal activity-inhibited activity)/(normal activity)]×100%

### 3.10. Determination of Superoxide Dismutase Activity

The activity of superoxide dismutase was determined as described by Misra and Fridovich [52]. The assay mixture contained 0.5 mL of hepatic PMS, 1 mL of 50 mM sodium carbonate, 0.4 mL 25 μm nitroblue tetrazolium and 0.2 mL of freshly prepared 0.1 mM hydroxylamine hydrochloride. The reaction mixture was mixed quickly by inversion followed by the addition of the clear supernatant of 0.1 mL of liver homogenate (10% *w*/*v*). The change in absorbance was recorded at 560 nm. The percentage of inhibition was calculated using this Equation:

(2) % superoxide dismutase inhibition=[(normal activity-inhibited activity)/(normal activity)]×100%

### 3.11. Estimation of Malonyldialdehyde (MDA)

The method described by Okhawa *et al*. [53] was employed to determine the level of lipid peroxidation in the animal tissue. Briefly, the reaction mixture of 0.2 mL of 8.1% sodium dodecyle sulfate, 1.5 mL of 20% acetic acid solution adjusted to pH 3.5 with sodium hydroxide and 1.5 mL of 0.8% aqueous solution of thiobarbituric acid was added to 0.2 mL of 10% (*w*/*v*) of the homogenate. The mixture was brought up to 4.0 mL with distilled water and heated at 95 °C for 60 min. After cooling with tap water, 1.0 mL distilled water and 5.0 mL of the mixture of *n*-butanol and pyridine (15:1 *v*/*v*) was added and the mixture centrifuged at 2000 rpm for 10 min. The organic layer was removed and its absorbance measured at 532 nm and compared with those obtained from MDA standards.

### 3.12. Histopathological Analysis of the Liver and Aorta

The, liver and aorta of the animals from each group were fixed in 10% formaldehyde, dehydrated, and paraffin blocks prepared for histopathological examination. The block was sectioned at 5–7 μm and stained with heamotoxylen.

### 3.13. Statistical Analysis

All data were expressed as mean ± SD of six replicates and were subjected to one-way analysis of variance (ANOVA) followed by Duncan multiple range tests to determine significant differences in all the parameters. Values were considered statistically significant at *p* < 0.05.

## 4. Conclusion

The present study showed that the methanolic extract of TVR produced a protection against atherogenic diet induced aortic pathology, enzyme depletion glomerulosclerosis and hepatic damage by preventing hyperlipidemia and oxidative stress in rats.

## Figures and Tables

**Table 1:** Food intake, body weight and percentage of weight gain of experimental groups.

Treatment	AIW (g)	AFW	% weight gain	FI (g/2 weeks)
Control	151.0 ± 0.11	208.0 ± 0.14	38.0	341.11
Athero-diet	152.1 ± 0.12	237.1 ± 0.12 *	55.9	353.62
Athero-diet + TVR (250 mg/kg)	150.2 ± 0.10	215.2 ± 0.11	36.7	346.24
Athero-diet + TVR (500 mg/kg)	153.0 ± 0.13	210.3 ± 0.13	34.6	343.40
Athero-diet + TVR (30 mg ator)	152.4 ± 0.12	209.1 ± 0.11	37.2	342.21

Ath-diet represents an atherogenic diet, TVR represents *Tulbaghia violacea* and Ator represent atorvastatin, AIW represents the average initial weight, FW represents the average final weight and FI represents food intake.

* represents a significant difference (*p <* 0.05) from the control.

**Table 2:** Effect of methanolic extract of *Tulbaghia violacea* rhizomes (TVR) on serum lipid profile in diet-induced atherogenic rats.

Treatment (mg/kg)	TG (mg/dL)	TC (mg/dL)	LDL (mg/dL)	VLDL (mg/dL)	HDL-c (mg/dL)	Atherogenic index (AI)
Control	63.32 ± 0.25 ^a^	75.11 ± 0.21 ^a^	7.45 ± 0.23 ^a^	15.94 ± 0.50 ^a^	39.10 ± 0.41 ^a^	0.92 ± 0.12
Ath diet	99.3 ± 1.23 ^b^	148.3 ± 0.23 ^b^	25.24 ± 0.17 ^b^	38.12 ± 0.19 ^b^	11.59 ± 0.35 ^b^	11.80 ± 0.15
TVR (250)	79.35 ± 0.43 ^c^	95.21 ± 0.22 ^c^	19.18 ± 0.15 ^c^	25.43 ± 0.23 ^c^	21.61 ± 0.27 ^c^	3.41 ± 0.14 ^a^
TVR (500)	71. 78 ± 0.21 ^c^	83.11 ± 0.25 ^d^	10.44 ± 0.21 ^d^	19.25 ± 0.14 ^d^	29.41 ± 0.19 ^d^	1.83 ± 0.11 ^b^
Ator- (30)	66.16 ± 0.24 ^a^	80.34 ± 0.31 ^d^	8.54 ± 0.32 ^d^	16.19 ± 0.11 ^d^	31.29 ± 0.16 ^d^	1.56 ± 0.13 ^c^

Values are Mean ± SD; *n* = 6, values with different superscripts (a, b, c, d) in the same column is significantly different from the normal control (*p* < 0.05) or atherogenic control group (Ath diet). TVR represents *Tulbaghia violacea rhizome*, Ath diet represent atherogenic diet and Ator-represent atorvastatin.

**Table 3:** Effect of TVR extract on markers of endothelial dysfunction in diet-induced atherosclerogenic rats.

Group	PLT (g/L)	Fibrinogen (g/L)	NO (μmol/L)
Normal	405.2 ± 0.25 ^a^	1.95 ± 0.43 ^a^	57.33 ± 0.11 ^a^
Ath diet	847.4 ± 0.21 ^b^	3.21 ± 0.28 ^b^	46.38 ± 0.18 ^b^
TVR (250 mg/kg)	490.6 ± 0.23 ^a^	2.63 ± 0.25 ^c^	51.36 ± 0.25 ^a^
TVR (500 mg/kg)	420.4 ± 0.16 ^a^	2.01 ± 0.39 ^d^	53.28 ± 0.35 ^a^
Ato- (30mg/kg)	415.9 ± 0.51 ^a^	1.99 ± 0.16 ^d^	55.19 ± 0.22 ^a^

Values are mean ± SD; *n* = 6, mean values with different superscripts (a, b, c, d) in the same column are significantly different from the normal control (*p* < 0.05) and atherogenic control group (*p <* 0.05). TVR represents *Tulbaghia violacea rhizome*, Ath diet represent atherosclerotic diet while Ator- represents atorvastatin and PLT represents platelet counts.

**Table 4:** Effect of methanolic extract of TVR on serum markers of liver and kidney damage in diet-induced atherogenic rats.

Group	AST (IU/L)	ALT (IU/L)	ALP (IU/L)	LDH (IU/L)	T-Bilirubin (mg/dL)	Creatinine (μmol/L)
Normal	23.12 ± 0.15 ^a^	26.03 ± 0.13 ^a^	42.33 ± 0.17 ^a^	69.18 ± 0.11 ^a^	0.21 ± 0.21 ^a^	61.12 ± 0.50 ^a^
Ath diet	47.32 ± 0.21 ^b^	51.21 ± 0.18 ^b^	92.38 ± 0.14 ^b^	138.3 ± 0.23 ^b^	5.43 ± 0.25 ^b^	67.11 ± 0.32 ^b^
TVR (250 mg/kg)	38.61 ± 0.33 ^c^	41.13 ± 0.15 ^c^	58.22 ± 0.45 ^c^	98.12 ± 0.20 ^c^	3.98 ± 0.19 ^c^	65.01 ± 0.21 ^c^
TVR (500 mg/kg)	31.55 ± 0.16 ^c^	35.43 ± 0.19 ^d^	51.14 ± 0.31 ^c^	79.33 ± 0.35 ^d^	2.14 ± 0.25 ^d^	62.23 ± 0.38 ^a^
Ato- (30mg/kg)	28.89 ± 0.51 ^a^	34.63 ± 0.16 ^d^	50.69 ± 0.42 ^c^	73.41 ± 0.31 ^d^	2.11 ± 0.24 ^d^	61.99 ± 0.26 ^a^

Values are mean ± SD; *n* = 6, mean values with different superscripts (a, b, c, d) in the same column are significantly different from the normal control (*p* < 0.05). Ath diet represents an atherosclerogenic diet, TVR represents *Tulbaghia violacea* rhizomes while T-Bilirubin represents total bilirubin.
